# A spatial dissection of the Arabidopsis floral transcriptome by MPSS

**DOI:** 10.1186/1471-2229-8-43

**Published:** 2008-04-21

**Authors:** Jason A Peiffer, Shail Kaushik, Hajime Sakai, Mario Arteaga-Vazquez, Nidia Sanchez-Leon, Hassan Ghazal, Jean-Philippe Vielle-Calzada, Blake C Meyers

**Affiliations:** 1 Department of Plant and Soil Sciences, University of Delaware, Newark, DE 19711, USA; 2 DuPont Crop Genetics, Wilmington, DE 19880, USA; 3 National Laboratory of Genomics for Biodiversity and Department of Genetic Engineering, CINVESTAV Campus, Guanajuato, Irapuato, Mexico; 4 Department of Plant Breeding and Genetics, Cornell University, Ithaca, NY 14850, USA; 5 Department of Plant Sciences, University of Arizona, Tucson, AZ 85721, USA; 6 University Mohammed I, Laboratory of Genetics and Biotechnology, Faculty of Sciences, Oujda and Pluridisciplinary Faculty of Nador, Morocco

## Abstract

**Background:**

We have further characterized floral organ-localized gene expression in the inflorescence of *Arabidopsis thaliana *by comparison of massively parallel signature sequencing (MPSS) data. Six libraries of RNA sequence tags from immature inflorescence tissues were constructed and matched to their respective loci in the annotated *Arabidopsis *genome. These signature libraries survey the floral transcriptome of wild-type tissue as well as the floral homeotic mutants, *apetala1, apetala3, agamous*, a *superman/apetala1 *double mutant, and differentiated ovules dissected from the gynoecia of wild-type inflorescences. Comparing and contrasting these MPSS floral expression libraries enabled demarcation of transcripts enriched in the petals, stamens, stigma-style, gynoecia, and those with predicted enrichment within the sepal/sepal-petals, petal-stamens, or gynoecia-stamens.

**Results:**

By comparison of expression libraries, a total of 572 genes were found to have organ-enriched expression within the inflorescence. The bulk of characterized organ-enriched transcript diversity was noted in the gynoecia and stamens, whereas fewer genes demonstrated sepal or petal-localized expression. Validation of the computational analyses was performed by comparison with previously published expression data, *in situ *hybridizations, promoter-reporter fusions, and reverse transcription PCR. A number of well-characterized genes were accurately delineated within our system of transcript filtration. Moreover, empirical validations confirm MPSS predictions for several genes with previously uncharacterized expression patterns.

**Conclusion:**

This extensive MPSS analysis confirms and supplements prior microarray floral expression studies and illustrates the utility of sequence survey-based expression analysis in functional genomics. Spatial floral expression data accrued by MPSS and similar methods will be advantageous in the elucidation of more comprehensive genetic regulatory networks governing floral development.

## Background

The majority of genes implicated in floral development have been identified through characterization of mutants displaying severe phenotypic deviations from wild-type development. An interesting subset of these mutants is the group of homeotic floral phenotypes. In these mutants, the organs of a single whorl of the inflorescence are duplicated within another distinct whorl at the expense of the organs typically present. The premise for these mutations is explained in the classic "ABC model" of floral development for *Arabidopsis *[1,2]. According to the ABC model, interactions among MADS-box transcription factors including, but not limited to *APETALA 1 *(*AP1*), *AGAMOUS *(*AG*), and *APETALA 3 *(*AP3*) are required for sepals, petals, stamens, and gynoecia development. Functional absence of any of these transcription factors results in the homeotic replacement of floral organs. The "quartet model" of protein interactions explains the genetic ABC model [3] and proposes the formation of five distinct whorl-specific tetrameric complexes capable of binding DNA and activating downstream genes responsible for organ development through *cis*-regulation at dual CArG boxes [3]. *In vitro *analysis has revealed heterodimeric interactions among MADS-box transcription factors [4]. Furthermore, *in vivo *interactions of homologous petunia MADS-box proteins involved in a putative ovule-defining quaternary complex were also observed [5].

Despite structural support for the quartet model, many regulatory aspects of this model have yet to be identified. A number of inflorescence meristem-identity genes such as *LEAFY*, *UNUSUAL FLORAL ORGAN*, *LEUNIG*, and *CURLY LEAF *have been linked to the upstream regulation of the genes encoding these quaternary complexes. However, few downstream organ-specific genes directly activated by these complexes have been identified [6]. Moreover, downstream targets such as *FRUITFUL*, *SPOROCYTELESS/NOZZLE *and *NO APICAL MERISTEM *do not obey the single whorl premise of the quartet model. [7-9]. Characterization of organ-specific gene expression downstream of the putative quaternary complexes is necessary to validate its functionality and understand the nature of its targets.

Genomic approaches have become a valuable tool in characterizing organ-related gene expression and in elucidating the genetic networks of floral development at a global level. Genome-wide analyses of transcript enrichment among Arabidopsis floral organs have been performed with the aid of hybridization-based approaches such as cDNA and oligonucleotide microarrays [10-20] and represent a strong first step in spatial characterization of the floral transcriptome. However, microarray analyses and other hybridization-based approaches are subject to a number of inherent limitations, including sensitivity to RNA quantity, non-specific probe hybridization, and substantial background levels capable of masking transcripts with low expression rates [21]. Furthermore, quantitative analysis across multiple microarrays requires the standardization and calibration of chips to ensure equivalent hybridization.

Although technical improvements are addressing several of these microarray issues, signature sequencing (such as massively parallel signature sequencing, MPSS) represents an alternative to microarrays and can overcome a number of limitations inherent to hybridization-based technologies and other conventional methods of large-scale gene expression analysis. Developed at what is now Illumina, Inc. (originally Lynx Therapeutics, Hayward, CA), MPSS reactions permit the simultaneous or parallel sequencing of 17 or 20 nucleotide "signatures" corresponding to distinct cDNA molecules from a sample [22,23]. These expression signatures may then be matched to their corresponding sequence in the genome to delineate gene expression. The length of MPSS signatures usually permits a single match to the Arabidopsis genome and enables highly specific quantification of transcription [24]. The background level afforded by this technology is superior to the level of transcript detection permitted by hybridization-based technologies and enables detection of transcriptions with lower expression levels [21] such as many transcription factors. Also, the linear normalized nature of MPSS data acquisition reduces the importance of signal standardization between cDNA libraries. Nonetheless, previously described "bad words" in MPSS sequencing reactions [25], as well as the absence of the necessary restriction sites within a particular cDNA, dilution of transcripts with low expression in diverse transcriptomes, and the cost-prohibitive nature of replicates are shortcomings of the technology. Therefore, integration of microarray studies and MPSS in future meta-analysis will permit improved genome-wide expression characterization, as will advances in short read DNA sequencing technologies, such as Illumina's current "sequencing-by-synthesis" (SBS) method.

In this study, we have implemented MPSS to dissect those genes enriched within the petal, stamen, gynoecium inclusive and exclusive of the ovule, and those of the sepal/sepal-petal, petal-stamen, or stamen-gynoecium. MPSS signatures were matched to genomic annotations (TAIR Version 6) and integrated into our publicly available web interface [26]. Using the interface, expression patterns were cross-analyzed to dissect floral organ(s) of enriched expression. Wild-type inflorescences were used to characterize transcript expression from all floral organs. The mutant *agamous *("*ag*," SALK_014999), was incorporated to delineate a complete loss of stamen and gynoecia, due to the homeotic replacement of reproductive organs by perianth whorls. Conversely, a loss of perianth whorls was obtained through the use of *apetala 1–10 ("ap1")*, an apetalous mutant with increased reproductive whorls and secondary apetalous inflorescences homeotically replacing most sepals. A loss of the petals and stamen expression was achieved through use of *apetala 3–6 ("ap3")*, a mutant in which organs of the petal and stamen whorls have been completely replaced by carpels and sepals. Although reduced carpel tissue was present, the double mutant *superman-2 apetala 1–10 ("sup ap1") *was preferentially selected for carpel reduction and used to characterize stamen enrichment at the expense of all other floral organs. In addition to utilizing whole-inflorescence libraries to determine organ-enriched gene expression, dissected differentiated ovules, roots, and leaves were also used to determine the ovule inclusivity of gynoecia-expressed transcripts and enrich for floral expression on a plant-wide basis.

## Results and Discussion

### Comparison of inflorescence expression profiles

Sequence-surveys have become a widely implemented approach to determine expression patterns and further elucidate developmental processes on a genome-wide scale. To further characterize floral development, we have constructed MPSS signature libraries from cDNA of wild type, *ap1, ap3, ag, sup ap1 *inflorescences during the first twelve stages of development [27] as well as differentiated ovule, root, and leaf tissues. MPSS signatures were matched to their respective gene annotations to delineate active expression in a relative manner (measured in transcripts per million transcripts; TPM). The significant expression of 21,715 genes was characterized within the floral inflorescences of the genotypes examined. To enrich for flower-specific transcripts, MPSS leaf and root expression data were compared to inflorescence expression profiles. Only 7.9 to 13.4% of the inflorescence-expressed genes were not observed (i.e. 0 TPM) in the leaves or roots (Table 1). Therefore, the majority of genes identified in the inflorescence were not flower specific. Nonetheless, with the exception of *ap3*, the mean expression level of genes with non-floral specific spatial expression was significantly greater in all inflorescences than leaf or root tissue (p < 0.001).

**Table 1:** Inflorescence transcriptome diversity, as measured by MPSS

Floral Strain or Tissue	Total Distinct Expressed Genes^a^	Inflorescence Enriched (%)^b^	Undetected within Leaf or Root (%)^c^
wild type	14,338	49.46	12.63
*apetala 1*	14,918	47.51	13.38
*apetala 3*	14,431	51.74	10.61
*agamous*	12,026	50.19	7.93
*sup/ap1*	12,505	55.53	10.97
Ovule	10,897	58.97	9.65

^a ^MPSS signatures within exons, within 500 bp of an annotated gene, in an intron, and overlapping an exon-intron splice boundary were quantified and "insignificant" signatures in only one sequencing library and those signatures corresponding to multiple regions of the genome were removed to demarcate gene expression.

^b ^Enrichment within the inflorescence signifies only those genes expressed at >4 TPM within the inflorescence. Moreover, only those genes whose expression in root or leaf tissue was less than expression within the mutant inflorescence were considered inflorescence-enriched.

^c ^Genes with TPM >0 in MPSS root and leaf libraries were subtracted from each floral library to assess inflorescence-specificity.

The selection of inflorescence-specific expression using a 0 TPM threshold in leaves and roots was too stringent and resulted in a high false negative rate of identification when compared to several genes with known expression patterns (data not shown). Therefore, inflorescence-enriched gene selection was accomplished by removing genes with lower expression in inflorescences than root and leaf tissues. Furthermore, only those genes expressed in inflorescences at greater than 4 TPM were included in our filtering criteria (Table 1). Using even less stringent parameters, many floral organ enriched transcripts are accurately depicted, as evident in our biological validations; however, increasing this threshold reduced the false discovery rate and increased the informatics power to accurately detect floral expression enrichment. Trends in transcript diversity across inflorescence genotypes were not significantly impacted by inflorescence enrichment. Within both raw data and filtered inflorescence enriched expression data, the highest level of transcript diversity was observed in wild type, *ap1*, and *ap3 *inflorescences and the lowest in *sup ap1 *and *agamous *(Table 1). This is likely due to the increase in reproductive organs associated in the former, and significantly decreased floral organ diversity and strictly vegetative organs in the latter. Although differences in transcriptome diversity between these groups were significant, differences in diversity among inflorescences within both groups were not statistically significant (p > 0.10).

After enriching for inflorescence expression, the expression profile of each MPSS library was characterized to reveal relationships between gene expression and phenotype and delineate possible biases among the data. The conservation of gene expression was determined across all libraries on a library intersection and a library relative basis. In our intersection approach, the conserved genes in two inflorescence enrichment filtered libraries were compared to the number of genes within both libraries. For example, (*ap1 *∩ *ap3*)/(*ap1 *∪ *ap3*) reveals a normalized correlation based on the presence or absence of genes within our filtering criteria (Table 2). Our library relative approach was based on calculating genes without statistically significant differences (p < 0.001) in inflorescence expression levels (as measured in TPM) within the two libraries. The number of genes not statistically different was normalized by the total distinct genes expressed in the two libraries being compared to develop a proportion (Table 2). Both methods revealed similar results for libraries possessing significantly different correlations.

**Table 2:** Correlation of expression across inflorescence libraries

Library intersection based comparison (%)^a^				
Inflorescence	wild type	*apetala 1*	*apetala 3*	*agamous*

*apetala 1*	82.05			
*apetala 3*	78.53	80.12		
*agamous*	72.45	72.32	75.32	
*sup ap 1*	73.92	74.44	71.36	68.86

Library relative based comparison (%)^b^				

Inflorescence	wild type	*apetala 1*	*apetala 3*	*agamous*

apetala 1	75.61			
apetala 3	72.82	75.51		
agamous	72.30	70.81	70.69	
sup ap 1	59.33	61.93	59.54	57.23

^a ^Intersection analyses was based on the presence or absence of genes within the library (library 1 ∩ library 2)/(library 1 ∪ library 2) after filtering the libraries for inflorescence enrichment.

^b ^Relative analyses was based on normalizing those genes without significant expression differences (p < 0.001) by the union of the two libraries under analysis.

The most similar expression patterns were obtained from *ap1 *and wild type inflorescences. The homeotic mutants *ap1 *and *ap3 *as well as *ap3 *and wild type were also highly correlated (Table 2). The difference between the three correlations was statistically significant (p < 0.001) and their relative rank was conserved in both the library intersection and relativity based approaches. Therefore, increases in gynoecium biomass at the expense of perianth tissue did not alter gene expression as much as the loss of reproductive organs. Moreover, the largest phenotypic differences were mirrored by the most significant differences in gene expression. The increase of stamens within the inflorescence of *sup ap1 *produced the greatest difference when compared to the strictly vegetative *agamous *inflorescences within both methods of comparison despite similar degrees of transcript diversity within the two libraries.

To identify additional alterations in expression in *ap1 *generated by the addition of the *sup *mutation and to address possible phenotypic correlations, the expression profile of *sup ap1 *and *ap1 *were compared. Although the proportion of conserved genes between *sup ap1 *and *ap1 *was greater than that of *sup ap1 *and wild type inflorescences, this difference was not significant (p > 0.05) within the library intersection analysis. In the library relative analysis, the similarity of gene expression among the *sup ap1 *and all other inflorescences was relatively low (Table 2). This suggests that increases in stamens at the expense of all other tissues altered the inflorescence expression profile more than any other mutation. Despite the substantial gene expression differences, *ap1 *revealed the most similar inflorescence expression pattern to *sup ap1 *in the library relative analysis (p < 0.001).

### Identification of organ-enriched gene expression

To identify transcripts highly enriched within a floral organ and reduced in other organs, we analyzed the data using a binary system. The occurrence of a specific organ or group of organs within a single floral strain was characterized as a "1" if present and a "0" if absent. Genes with an expression pattern mirroring the organ occurrence profile across the homeotic mutants (Table 3) were identified as enriched for the respective organ. For example, stamens are present within *ap1 *and *sup ap1*; however, they are absent in *ap3 *and *agamous*. Therefore, only those genes expressed at greater than 4 TPM within *ap1 *and *sup ap1 *and absent (0 TPM) from *ap3 *and *ag *MPSS libraries were deemed "stamen-enriched". To further filter for floral organ-enrichment on a plant-wide basis, only transcripts expressed at a greater rate (TPM) within the inflorescence than leaf or root tissue were included in the analyses.

**Table 3:** Organ occurrence profiles

MPSS Library				Putative organ(s) of expression
	
*ap1*	*ap3*	*ag*	*sup ap1*	
0	0	1	0	Petal
1	0	0	1	Stamen
1	1	0	0	Gynoecium
1	1	0	1	Stamen-Gynoecium
1	0	1	1	Petal-Stamen
0	1	1	0	Sepal-Petal, Sepal
1	1	1	0	Sepal-Gynoecium, Sepal-Petal-Gynoecium, Petal-Gynoecium
1	1	1	1	Sepal-Stamen, Petal-Stamen-Gynoecium, Sepal-Stamen-Gynoecium, Sepal-Petal-Stamen, All floral organs

"1" is representative of the respective organ(s) presence within the respective homeotic mutant, and "0" represents the organ(s) absence/diminishment. Organ occurrences were matched to transcript expression levels "1" = leaf or root expression < transcript expression and >4TPM, "0" = transcript expression = 0 TPM to determine organ enriched gene expression.

The ability to dissect all possible patterns of floral organ expression is limited by the diversity of the mutant libraries employed within this study (Table 3). Enriched expression within an organ or multiple organs may only be recognized if their respective organ occurrence profiles are unique. For example, sepal-enriched transcripts cannot be isolated from sepal and petal expressed transcripts, because transcripts from both spatial enrichment groups are present or up regulated within *agamous *and *apetala3 *mutants and down regulated within *ap1 *and *sup ap1*. Similar limitations were inherent within previous homeotic microarray analyses and likely led to the characterization of *Apetala1*, a sepal and petal expressed transcript [28], as expressed in a sepal-specific manner within the previous data set [10]. In addition, organ specific prediction may be biased by additional alterations in transcription as a result of homeotic transformation. An example of this is the dependence of sepal expression on the co-existence of sepals and petals. Putative petal-enriched transcripts may also be expressed within the sepal in wild type inflorescences but not in *apetala3*; the mutant used to distinguish a lack of expression in the sepal and gynoecium. Bearing in mind these limitations, the presence of transcript enrichment within the organ(s) of inflorescence may only be dissected for seven instances within our MPSS analysis: sepal/sepals-petals, petals, stamens, petals-stamens, stamens-gynoecia, and gynoecia inclusive and exclusive of the ovules (see Additional file 1 for a complete list of transcripts).

**Figure 1:**
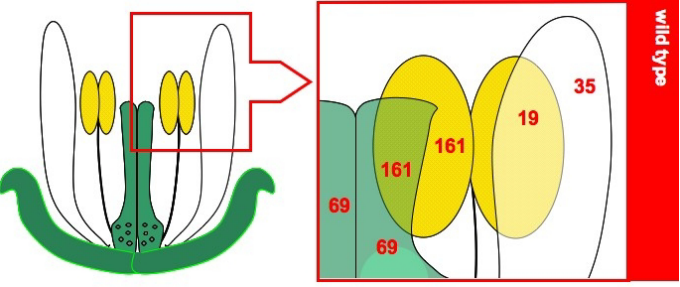
**Transcriptional diversity of floral organs, as predicted by MPSS**. The number of genes matching our stringent filtration of MPSS expression libraries for each organ-enriched set of genes.

Figure 1 reveals the number of genes identified as enriched within the floral organ(s) under analysis. The number of genes enriched within each floral organ has likely been underestimated in order to reduce our false discovery rate; however, the relative trends in transcript diversity that we identified were maintained even under relaxed filtering parameters based merely on gene presence (>0 TPM) or absence (0 TPM). The greatest transcript diversity was noted within the reproductive organs, whereas much less active expression occurred in vegetative tissues or within both the stamens and the petals.

A total of 138 genes were identified as possessing gynoecium-enriched expression within our analyses. This ranks in close proximity to the 161 genes characterized within the stamens and the 161 genes identified as expressed throughout both reproductive organs. The level of transcript diversity noted within the gynoecium was similar irrespective of the ovule; both gynoecium expression inclusive of the ovule and exclusive of the ovule identified a total of 69 genes. This suggests during the first twelve stages of floral development [27], the ovule does not express from an extensively larger subset of gynoecium-enriched genes than the stigma, style, the placenta, or carpel wall. Despite the extensive diversity noted in previous microdissection studies [16], only 35 genes with petal-enriched expression were identified in *agamous *but absent from other mutant inflorescences. Nonetheless, this was greater than the 19 transcripts identified as expressed within both the stamen and the petals. Therefore, fewer transcripts are likely to be enriched across only the petals and stamens than are specifically enriched in each organ. This contrasts with the trend noted across the reproductive organs wherein nearly equivalent numbers of genes were identified as enriched within a single organ as expressed throughout both reproductive tissues. Unlike previous comparisons that enabled dissection of enrichment within a single organ or a cluster of two organs, the 56 genes enriched within the sepal and throughout the perianth could not be dissected from each other given the mutants employed in this study. Therefore, the relative transcriptional complexity of the sepals cannot be compared to the petals. Nonetheless, it can be concluded that the number of sepal-enriched genes does not supersede those identified within any of the reproductive organs.

**Figure 2:**
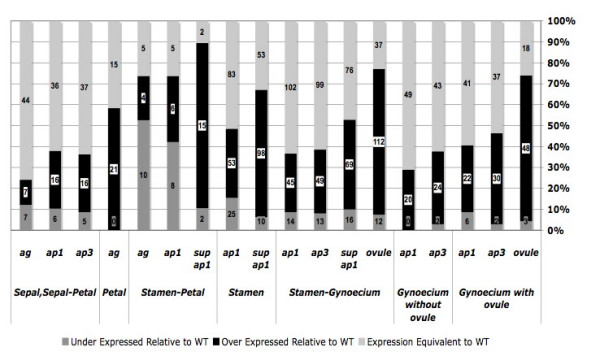
**Expression of genes with putative organ-enriched expression relative to wild type in homeotic mutants with expected over expression**. Homeotic mutant expression levels of genes with organ-enriched expression were compared to wild type expression. The proportion of genes over-expressed (p < 0.05), under-expressed (p < 0.05), and not significantly different (p > 0.05) relative to wild type were determined within each organ-enriched gene set and homeotic mutant of expected over expression. Values on bars refer to the number of genes within each data set.

After determining the relative diversity of the predicted organ-enriched gene sets as measured by MPSS, gene expression levels were compared. Given that homeotic mutants with increased development of a floral organ should express genes enriched within that organ at a significantly higher level than wild type, each organ-enriched gene set was analyzed to identify the proportion of genes showing significant over-expression within the mutant tissue relative to wild type. This approach approximates that method previously employed using microarray data; nonetheless, more significant overlap was noted between previous microarray analysis and our threshold-based MPSS methods than MPSS data analysis based on expression relativity to wild type. The proportion of organ-enriched genes differentially expressed relative to wild type was determined (Figure 2, Additional file 1). The mean proportion of organ-enriched genes showing differential expression (p < 0.05) was 65.10% ± 24.12% (95% C.I.). Of the differentially expressed genes, the majority were over-expressed within their expected mutants, 81.20% ± 19.77% (95% C.I.). Therefore, our analysis does not substantially increase the number of genes detected that reveal significant increases relative to wild type inflorescences. However, those floral organ-enriched genes showing differential expression are largely over-expressed in the mutants that accumulate biomass of the respective organ (Figure 2).

To further characterize the expression patterns of our organ-enriched gene sets, the temporal expression of our spatial gene sets was determined. Although little commonality was noted between independent microarray analyses of spatial Arabidopsis expression and our organ-enriched gene sets, the spatial data accrued from this study was superimposed on cumulative temporal data identified in the GENEVESTIGATOR online microarray database to delineate the temporal expression of our organ-enriched gene sets [20]. The majority of genes with MPSS determined floral organ-enriched expression were detected in later stages of floral development after 10% of the flower buds have opened (>36 days after planting). Therefore, our organ-enrichment analyses may be skewed to detect more genes expressed near stage twelve of development, after significant biomass for all floral organs has developed.

### Functional characterization of organ-enriched gene sets

Genes possessing predicted organ-enriched expression were further characterized based on gene ontology and *cis*-element conservation to further understand the nature of those factors underpinning organogenesis and the maintenance of specific floral organs, as well as to identify the relative impact of the quaternary complex in activating organ-enriched genes. Regulation and function were determined using several publicly available data mining tools. The abundance of organ-enriched genes with common regulators or function was compared to the mean genome-wide level of expression to identify overrepresented characteristics within genes associated with each floral organ.

Delineation of gene function enrichment was achieved using the publicly available analysis tool EasyGO [29]. An enrichment of terms detailing specific molecular functions, as well as associations with cellular components were identified within gene sets. Few enriched terms were identified in the majority of organ-enriched gene sets, genes possessing stamen-enriched expression were enriched for lipase activity (p < 0.01) as well as presence within the endo-membrane system (p < 0.01). The lipase *DEFECTIVE ANTHER DEHISCENCE *has been shown to catalyze the initial step in the jasmonic acid pathway that regulates flower opening, synchronization of pollen maturation, and anther dehiscence [30]. Therefore, it is possible additional lipase genes are involved in similar pathways to regulate stamen maturation.

To assess the role of the MADS-box proteins involved in the quaternary complex at regulating organ-enriched gene expression, several algorithms were implemented to identify overrepresented *cis*-elements within each set of genes. The upstream and downstream regions surrounding each gene were scanned for over-expressed motifs at 500 and 1000 bp intervals using The University of Toronto's Promoter 2 program [31] as well as the University of Leeds' and the Arabidopsis Gene Regulatory Information Server's, Known *Cis*-element Analyzer [32]. No significantly overrepresented motifs were characterized within any of the sets of genes with floral organ-enriched expression. This confirms previous microarray functional assessments and reveals no significant enrichment of CArg boxes in organ-enriched gene sets. Therefore, much of the noted organ-enriched expression is not mediated by a single common *cis*-regulatory element such as the action of the putative quaternary complex on CArg boxes [3] during later stages of floral development. Nonetheless, the expression of over 200 miRNAs has been characterized within wild type floral inflorescence using MPSS [33] and it is probable post-transcriptional regulation plays a substantial role in restricting organ-enriched expression in a manner similar to that noted between miR172 and *AP2 *[27]. Future analyses characterizing miRNAs in inverse expression patterns to those identified in Table 3 may enable detection of those miRNAs negatively regulating the expression of genes responsible for floral organ maintenance and development.

### Validation experiments I: MPSS correspondence with microarray analyses

The data sets produced using the threshold filtration approach of MPSS data were compared to several previous microarray studies to reveal the concurrence of analyses. Previous analysis comparing homeotic mutant gene expression relative to wild type revealed significant overlap. Approximately 70% (111 genes) of those genes identified by MPSS to be stamen-enriched agreed with this previous microarray analysis; however, the number of putative stamen-enriched genes identified within the previous study was over seven-fold greater than that identified by our analyses. This difference may be due to the difference in filtering stringency and not a discrepancy in the actual number of stamen-enriched genes. MPSS analyses were set to identify only the most probable candidates for organ enrichment and likely possess a high false negative rate. Relaxed MPSS filtration criterion based only on the absence of expression within mutants lacking stamens revealed a similar number of stamen-enriched genes. However, the fraction of organ-enriched genes identified under relaxed parameters that concurred with the previous analyses was greatly reduced to approximately 26% (267 genes).

Microarray analysis from isolated floral organs [16] and pollen [34] revealed an overlap of 25 genes within the stamen-enriched data set. Moreover, all 25 stamen-enriched genes identified through the intersection of MPSS and the dissected floral organ microarray results [16] concur with previous microarray analyses based on comparisons of homeotic mutants [10]. This is significantly greater than the proportion of stamen-enriched genes identified as common to both microarray studies alone (approximately 49%). Several known stamen-enriched and *Apetala3 *regulated genes, including *Profilin5*, *Arabinogalactan6*, *Cyp703A2*, *Anther7*, *Callose Synthase5*, *Aborted Microspore*, *Taptetum1*, *Lipid Transfer12*, and *Anther27*, were identified across the microarray and MPSS analyses. However, nearly half of the stamen-enriched genes identified across all studies have putative or unknown functions including genes related to a putative self-incompatibility gene, At5g26060, a putative phospholipase A2, At4g29470, involved in the jasmonic acid pathway regulating pollen maturation and several genes within the glycine-rich protein family which is known to possess high expression within the androecium.

Much less commonality was noted among organ-enriched gene sets of the gynoecium, petals, and sepal-petals (see Additional file 1). Only 15% (10 genes) of gynoecium-enriched genes as determined by MPSS were identified within previous microarray organ-enrichment studies [16]. Nonetheless, a few genes with known gynoecium-enriched expression were characterized by MPSS that were not identified in previous microarray analyses, including three *Embryo Defective *and *Embryo Defective*-like genes. MPSS-identified petal-expressed gene sets shared only two genes with microarray analyses [16]. Similarly, among the set of sepal/sepal-petal localized transcripts only five genes were identified as common to microarray analyses and MPSS predictions [10,16].

The significant reduction in similarity among MPSS and microarray [10] predicted genes within the gynoecia and vegetative organ-enriched expression suggests comparison of homeotic mutants is less accurate in determining enrichment of genes within these organs than the stamens in one or both analyses. However, previous microarray analysis detected significantly more genes with enriched expression in the stamens than the gynoecia despite the relatively similar levels of reproductive organ transcriptome diversity noted in this study.

### Validation experiments II: *In situ *hybridizations

**Figure 3:**
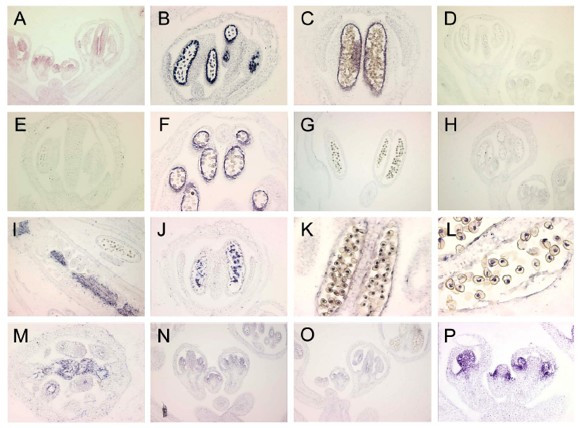
**Empirical validation via *in situ *hybridization**. **(A-C) **At1g33430 (anti-sense probe), (**D-E**) (sense control probe) noted signal on stamen and carpel primordial with strong expression detected in the tapetum and microspore as predicted by MPSS. **(F-G) **At2g19070 (anti-sense probe), (**H) **(sense control probe) signal identified on the tapetum. Stamen and petal localized expression predicted by MPSS **(I-J) **At1g54860 (anti-sense probe) signal on stigmatic papillae as well as septum and developing microspore. Predicted to be stamen-enriched by MPSS analysis. **(K-L) **At5g59810 (anti-sense probe) transient signal noted on a specific microspore of the tetrad. Carpel-enrichment predicted by MPSS. **(M) **At1g72290 (anti-sense probe) signal identified on the septum. Predicted to be localized within the stamen and the carpel by MPSS. **(N) **At2g42940 (anti-sense probe) **(O) **(sense control probe) weak signal on stamen and carpel primordial. Identified as a putative stamen-enriched transcript by MPSS. **(P) **AP3 (anti-sense control probe).

The previously uncharacterized expression of putative organ-enriched genes identified under our stringent and more relaxed (presence vs. absence) parameters were empirically validated through *in situ *hybridization. Expression of the galactosyltransferase family protein, At1g33430, was correctly assigned by relaxed MPSS criteria with expression in the stamen and carpel primordia and strong expression specifically within the tapetum and microspores [Figure 3a–c (anti-sense) 3d,e (sense)]. Our relaxed filtering system of MPSS data predicted At1g72290 to be expressed in both the gynoecia and stamens. However, *in situ *hybridization has only revealed expression within the carpel [Figure 3m (anti-sense)] and no expression was noted within the stamens. Similarly, At2g19070 is predicted by relaxed MPSS parameters to be localized within the petal-stamen. *In situ *hybridization patterns revealed its presence within only the tapetum; however, given the low level of MPSS predicted expression within the petal-enriched mutants, stringent filtering removed the transcript from the expression set [Figure 3f–g (anti-sense), 3h (sense)]. Using relaxed parameters, a putative stamen-enriched gene, At2g42940, was found expressed in both carpel and stamen primordium [Figure 3n (anti-sense), 3o (sense)]. Therefore, some genes enriched for the stamen or gynoecium may be expressed within both reproductive organs; however, it is less likely they are present within vegetative tissue as well.

Within the more stringent filtering parameters used to delineate organ-enriched gene sets, the expression of a gene with unknown function, At1g54860, was characterized as stamen-enriched. *In situ *hybridization revealed strong expression within the microspore as well as lesser expression within the stigmatic papillae and septum [Figure 3i–j]. This implies increased the filtering parameters may increase the accuracy of analysis for organ enrichment. However, a discrepancy between stringent MPSS and *in situ *data was noted in the putative gynoecium-enriched At5g59810. This gene encodes a subtilase family protein and was identified strictly within the stamen in a single microspore of the tetrad by *in situ *hybridization [Figure 3k–l]. Nonetheless, previous publications have revealed stigma-enriched expression of At5g59810 via several microarray analyses, RNA gel blots, and *in situ *analysis.

### Validation experiments III: GUS histochemical assays

**Figure 4:**
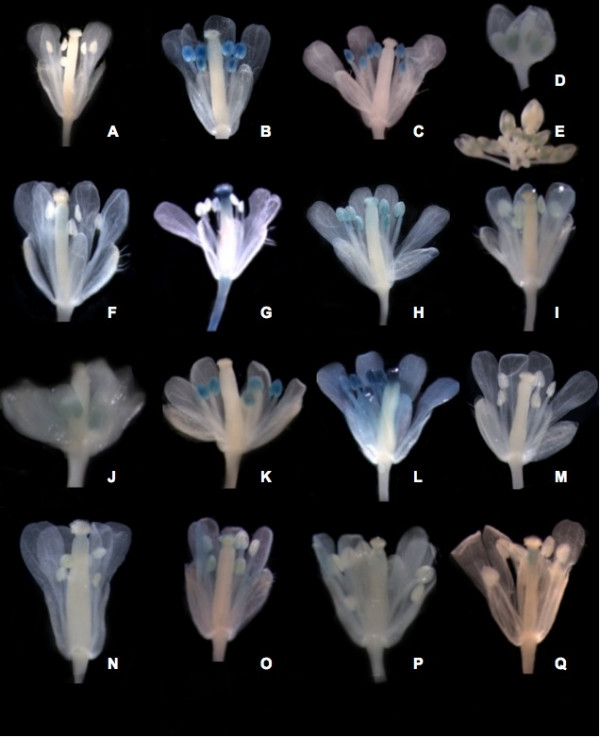
**Empirical validation by promoter:GUS fusions**. **(A) **Wild type control. **(B-E) **At1g20130 **[B]**, At3g27025 **[C]**, At2g42940 (**[D] **and **[E]**). MPSS-predicted stamen-enriched promoter:*GUS*. **(F-G) **At1g07370 **[F]**, At1g27900 **[G]**. MPSS-predicted carpel-enriched promoter:*GUS*. **(H) **At1g33430. MPSS-predicted stamen/carpel-enriched promoter:*GUS*. **(I-J) **At5g07550 **[I]**, At2g19070 **[J]**. MPSS-predicted stamen/petal-enriched promoter:*GUS*. **(K) **At1g07930. MPSS-*ap3 *expression enriched relative to other mutants. **(L) **At2g35340. MPSS-predicted absence of floral expression. **(M) **At1g26270. MPSS-predicted ubiquitous floral expression. **(N) **At1g68200. MPSS-*ap1 *sole expression. **(O) **At2g43100. MPSS-*ap3 *expression enriched relative to other mutants. **(P) **At3g15160. MPSS predicted absence in *sup ap1 *mutant. **(Q) **At52900. MPSS-*ap3 *and *ag *enriched expression.

Transgenic GUS reporter lines were used as another means of assessing the accuracy of floral organ localization analysis. A 1.5 kb region upstream of several genes with putative organ-enrichment was used to drive the expression of the GUS reporter gene. Promoter: GUS constructs with two or more independent transformation events were characterized via histochemical assay. No differential spatial expression was noted between transformation events of the same construct. The majority of organ-enriched expression predicted by MPSS using relaxed filtering parameters was mirrored in the GUS-staining activity of the promoters under analysis (Figure 4). Putative stamen-enriched expression was evaluated by promoter fusion in three genes. The promoters of At1g20130, At2g42940, and At3g27025, were all found to stain the stamen as predicted (Figure 4b–e). However, in the case of At2g42940, *in situ *analysis revealed additional expression in the carpel primordia. Two genes with putative gynoecia enriched expression were assayed as well. After staining, the promoter: GUS fusions for At1g07370 and At1g27900 were found to stain within the gynoecia as predicted (Figure 4f–g). MPSS predicted At1g33430 to be expressed in both reproductive organs. This result was substantiated through the histochemical assay, with staining occurring in both the stamens and gynoecia (Figure 4h). Two additional genes predicted with MPSS to have expression in both the stamen and petal were also assessed. Confirming our previous *in situ *hybridization results, At2g19070 was found expressed only in the stamens (Figure 4i). Despite this discrepancy, expression of At5g07550, a gene encoding a glycine-rich oleosin protein (GRP19) found to regulate the size and character of lipid droplets within the pollen coat [35] was demonstrated within both the stamens and the petals as predicted (Figure 4j). Expression of this gene in the petal has not previously been documented in the literature and may indicate additional uncharacterized activity; five independent transformation events revealed similar expression patterns (data not shown). At1g26270, was present in all floral MPSS libraries and, as expected for the promoter:GUS fusions, staining was prevalent throughout all floral organs (Figure 4l). Similarly, At2g35340 was assayed and the result confirmed MPSS predictions of null expression within the inflorescence tissues, (Figure 4m) despite previous microarray characterizations within floral tissues [20].

Although not present within our MPSS filtration parameters, several genes with expression enriched in a specific mutant relative to the other floral mutants were also characterized as controls for our filter parameters and the MPSS methodology. Contrary to MPSS predictions, At1g07930, a gene with very weak expression of 5 TPM in the stamen-less *ap3 *mutants and no expression within the stamen-enriched *sup ap1 *mutant was identified within the stamens (Figure 4k). Expressed in carpel- and stamen-enriched *ap1 *material but absent from all other floral mutants, At1g68200 was found present within the carpel as expected (Figure 4n). The *ap3 *enriched At2g43100 was found to be expressed in the carpel and petals despite the nearly-undetectable expression level of only 1 TPM in the highly petal-enriched *agamous *mutant (Figure 4o). At3g15160 was identified by MPSS as expressed throughout all floral organs except *sup ap1 *at a level higher than 10 TPM; experimentally, expression was detected in the petals and gynoecia but not within the stamens (Figure 4p). GUS staining demonstrated carpel-specific activity for At3g52900, and it was also identified as highly expressed within the carpel-enriched *ap3 *by MPSS analysis although significant expression (9 TPM) was also noted in the carpel-devoid *agamous *mutant. These results suggest absence or presence of expression within a single mutant was not as accurate at detecting expression patterns in the organs of enrichment; however analysis across all four mutants improved detection abilities.

**Figure 5:**
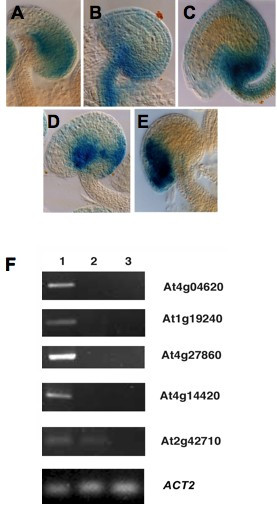
**Expression patterns conferred by promoter:uidA fusions and reverse transcription PCR corresponding to genes with enriched ovule expression**. Promoter:*uidA *fusions. Absence of GUS expression in additional floral organs (sepals, petals, stamens and gynoecia) was confirmed for all lines except for At2g47470 that shows expression in the carpel walls and stigma (data not shown). **(A) **At1g05550; GUS is expressed in both integuments and the nucellus, at the chalazal region. **(B) **At5g24420; GUS is expressed in the funiculus and both integuments throughout the ovule. **(C) **At5g49180; GUS is expressed in both integuments and the funiculus, but not in the female gametophyte. **(D) **At3g06240; GUS is expressed in the anatropus integumentary ridge and a small region of the dorsal outer integument. **(E) **At1g27330: GUS is expressed in the micropylar pole and both integuments. **(F) **RT-PCR expression of genes predicted to be specifically expressed in the ovule. Total RNA was extracted from individual floral organs and used for reverse-transcriptase PCR analysis. Lane 1: fully differentiated ovules; Lane 2: petals; Lane 3: sepals. Amplified fragment sizes: At4g04620 (231 bp); At1g19240 (250 bp); At4g27860 (235 bp); At4g14420 (209 bp); At2g42710 (223 bp).

In addition to whole gynoecium promoter:GUS fusions and mutant enrichment based comparisons, ovule expression was identified in five genes with ovule-inclusive, gynoecium enrichment as predicted by relaxed MPSS parameters. A gene of unknown function, At1g05550, was identified within the integuments (Figure 5a). At5g24420 was characterized within the funiculus and both integuments of the ovule, confirming MPSS-based predictions of ovule expression (Figure 5b). Expression of At5g49180 was found in both the integuments and the funiculus; however, it was not expressed within the female gametophyte (Figure 5c). Transcriptional activity of At3g06240, a gene encoding an F-box protein was identified in the anatropous integumentary ridge and a small region of the dorsal outer integuments (Figure 5d) but not in other fully differentiated floral organs (data not shown), confirming the ovule-enriched expression pattern predicted by our MPSS analysis. At1g27330 was identified as expressed within the micropylar pole and integuments of the ovule as well as the chalazal region of the nucellus (Figure 5e).

### Validation experiments IV: Reverse transcription PCR validation

Validation of MPSS organ-enrichment filters was performed in five putative gynoecia expressed genes by reverse transcription PCR (Figure 5f). Expression patterns were found to correlate with those predicted by MPSS. At4g04620, a gene encoding ATG8b, a microtubule associated protein involved in autophagy demonstrated expression in the ovule as predicted by MPSS, without any expression present within sepal or petal organs. This isoform of ATG8 has been previously characterized by microarray studies [36] which reveal the highest level of floral expression within petal tissue, followed by stamen, with lesser amounts in sepal and carpel tissue; however, MPSS analysis reveals gynoecia enriched expression inclusive of the ovule. MPSS predictions of gynoecia enrichment were confirmed by reverse transcription PCR. No expression was detected in petal or sepal tissues, suggesting that the distinctive alternative transcript of At4g04620 is specifically expressed in the ovule (V. Pérez-España and J-Ph. Vielle-Calzada, unpublished results). In addition, At4g27860, At1g19240, and At4g14420 were also found by reverse transcription PCR to demonstrate ovule expression. No sepal- or petal-expressed transcripts were noted within these analyses. In contrast to MPSS predictions, reverse transcription PCR revealed the expression of At2g42710, a putative structural constituent of the large ribosomal subunit in both ovule and petal tissues. These many empirical analyses suggest organ enrichment was accurately identified through our analyses; however, to delineate the specificity of expression to a single organ a meta-analysis approach is necessary to further filter transcripts and attain even more robust data sets.

## Conclusion

Our MPSS floral transcriptome analysis has dissected additional expression data to further corroborate and supplement existing spatial analyses of gene expression performed using microarrays. Numerous well-characterized genes with known expression patterns were accurately dissected as organ-enriched within our system of transcript filtration. Furthermore, the validation experiments were largely consistent with the MPSS expression data for several previously uncharacterized genes. In agreement with previous analysis [10], reproductive structures possess the most diverse and complex transcriptome when compared to vegetative tissue and no significant enrichment of CArG boxes was noted upstream or downstream of putative organ-enriched genes, suggesting that floral organogenesis and maintenance requires a multitude of signalling cascades as opposed to extensive direct *cis*-regulation by the quaternary complex. This spatial dissection of transcript expression will provide a valuable reference for future functional studies, and represents a source for developing floral organ-enriched promoters to drive transgene expression. In addition, these promoters could be coupled to a recently-described system for isolation of specific cell types by fluorescence-activated sorting [37], followed by more advanced methods of gene expression analysis such as Illumina's Sequencing By Synthesis; a tag-sequencing platform with ten-fold higher sensitivity than MPSS [38]. This will enable even more detailed spatial profiling of gene expression in future floral studies.

## Methods

### Plant materials, tissue collection, and nucleic acid isolation

All plant materials used for MPSS and reverse transcription PCR analyses were from *Arabidopsis thaliana *ecotype *Columbia-0*. Floral inflorescences were harvested from plants grown in Pro-mix soil in a growth chamber with 16 h of light for 5 weeks at 22°C with 60% humidity. These floral tissues included inflorescence meristems as well as floral buds corresponding to the first 12 stages of development [27]. Leaf and root tissues were obtained from the same plants grown in 16 h of light for 21 d under sterile conditions in vermiculite and perlite. An automated micro-aspirator that allows ovule isolation and harvesting was used to collect ovule samples (M. Arteaga-Vazquez, M. Arteaga-Sanchez M. and J-P. Vielle-Calzada, unpubl. results). Sepal and petal tissues utilized in reverse transcription PCR analysis were hand-dissected from *ag *inflorescences. All tissue samples were harvested less than 2 h after dark and frozen at -80°C prior to nucleic acid extractions. *A. thaliana *ecotype *Columbia-0 *plants utilized for transformation of promoter: GUS fusion plasmids were grown in 16 h of light for 5 weeks under the same conditions prior to floral inoculation with *Agrobacterium tumefaciens*.

Floral tissues utilized within the *in situ *hybridization validation were derived from *Arabidopsis thaliana *of the *Landsberg erecta *ecotype. This was primarily due to the increased size of floral inflorescences as compared to the *Columbia-0 *relative. Plants were grown in a growth chamber with light, temperature and humidity conditions similar to those implemented to grow the plants for the creation of the MPSS libraries.

RNA used for cDNA for MPSS, *in situ *hybridization, and RT-PCR validation was isolated using the TRIzol (Invitrogen) reagent and the manufacturer's protocol. Genomic DNA isolated for the amplification of promoter sequences was obtained using the DNeasy Minispin Column Extraction Kit (Qiagen).

### Signature sequencing and genomic correspondence

MPSS was performed as previously described [22,23]. Signatures for each floral library were produced in multiple sequencing runs and in two distinct types of sequencing reactions [22,25]; these sequencing runs and reactions were joined to compute a single normalized abundance for each signature observed in each of the floral, root, and leaf MPSS libraries [25]. All raw and normalized signature data have been made publicly available on the web interface [26]. These signatures were matched to their respective loci within the *A. thaliana *genomic sequence. Briefly, potential MPSS signatures were computationally derived from all possible *Dpn*II restriction sites (GATC) and 13 adjacent bases within the genome. Potential MPSS signatures located on the sense-strand corresponding to an exon, intron, exon-intron splice boundary, or present within 500 bp of the 3' end of an annotated ORF were matched with the empirically derived MPSS sequences to determine the expression level of the respective gene or pseudogene.

### MPSS library filtration, floral expression cross-analysis, and sorting

All MPSS libraries implemented within this study were filtered with a "reliability" filter in order to remove potentially erroneous signatures and distinguish a subset of valid expression levels. This filter eliminates all signatures identified within only a single sequencing run across all current expression libraries. Each tissue utilized within MPSS library corresponds to a minimum of four distinct sequencing runs. Therefore, signatures not identified within any other runs are likely resultant from random MPSS sequencing errors, which have been estimated to occur at a rate of ~0.25% per base [25].

Once reliable MPSS expression data was accrued, libraries corresponding to floral tissue designated with "1" (organ presence) or "0" (absence) to demarcate the organ occurrence profile among the homeotic floral mutants (Table 3). In order to isolate the subset of genes expressed specifically within a given organ, the normalized signature data corresponding to each gene's expression was matched to each organ occurrence profile through the use of our publicly available library analysis (LIBAN) interface. Genes with floral organ-specific expression in a mutant lacking that specific floral organ(s) logically possesses a normalized transcription level of 0 transcripts per million assayed (TPM). In contrast, those homeotic mutants that possess the specific floral organ consequently express the transcript at a normalized rate of greater than 4 TPM and expressed at a greater rate within inflorescence libraries than leaf or root MPSS libraries.

Once a subset of genes with organ(s) specific expression was identified, they were further sorted by their level of expression within the mutants which most overexpresses the specific organ. Although this biases the data for genes of higher expression, this permitted the establishment of a relative degree of confidence in the MPSS prediction of organ-specific expression. For example, putative stamen-specific transcripts were sorted first based on their level of expression within the *superman/ap1 *mutant, then by their level of expression within *ap1 *(which also overexpresses stamens) and finally by the wild-type expression level.

In order to identify the genome-wide correlation of expression across the floral tissues assayed within this study in a relative and intersection based manner, the proportion of genes not revealing significant (p < 0.05) alterations in expression relative to the other library was normalized by the union of the two library transcriptomes. From a more qualitative threshold based prospective, genes identified as present within each library based on our threshold of 4 TPM and enrichment in the inflorescence were normalized by the union of the inflorescence libraries. Over or under expression relative to wild type was determined using a normal approximation test for difference (Z-test) in the binomial proportions of transcripts per million [39]. This analysis was performed using Microsoft Excel and SAS JMP v. 7.0.

### *In situ *hybridizations

*In situ *hybridization was performed on sections of *Landsberg erecta *(ler) floral tissues. Tissue fixation, sectioning and embedding were performed using a modified protocol [40]. Anti-sense probes and control sense probes were derived from the following regions of the genome: At1g33430 (5'-CGGGGAAAGCCATAATAGTGC---CAACAGCAACTGCAATG-3'); At2g19070 (5'-AAAAAGAAAGGGGGTTTGTGTT---ACTTCGGCAATGCTACTCTTG-3'); At1g54860 (5'-TGCCTATCGCTTAATTCTGCTT---GATTGTTGTGTTTTTGTGTGAA-3'); At5g59810 (5'-GACGTAAGCCCATGGTTGATGA---CGTTAGGAGTCCCATCGTCGTC-3'); At1g72290 (5'-GAGAGTAAAAACGGAGGTGGTC---CTTGAGAAAACATTGATCA-3'); At2g42940 (5'-TGTTGCAGGTACAAACTACAAA---ACAGGGACCAGATGCGATTAG-3'). These regions were amplified and cloned into pGEM-T; after which, their orientation was determined by sequencing from the T7 and SP6 promoter sites. Digoxigenin probe synthesis, antibody detection and staining were done using the manufacturer's protocol (Boehringer Mannheim). The slides were dehydrated using ethanol and xylene, and were mounted in Per-mount (Fisher Scientific). Sections were photographed through a Leitz DRB (Leica, Wetzlar, Germany) light microscope using Kodak Ektachrome 160 ASA film.

### Promoter:GUS fusions and histochemical assays

To construct non-ovule promoter:GUS vectors, a ~1.5 kb promoter fragment upstream of each respective start codon was amplified from genomic DNA of *A. thaliana, Col-0*. Gateway Cloning Technology (Invitrogen) was implemented to insert the amplicon into pDONR221, and ultimately into the binary expression vector pK2GWFS7 wherein the promoter was used to drive the expression of the reporter gene beta-glucuronidase. Primers used in fragment amplification and plasmid recombination are: At1g20130 (S5'-GGGGACAAGTTTGTACAAAAAAGCAGGCTTAGGATTTATTGGTGTTTCTC-3' and AS5'-GGGGACCAGTTTGTACAAGAAAGCTGGGTTGGCCACGGCTGTGGATACG-3'); At3g27025 (S5'-GGGGACAAGTTTGTACAAAAAAGCAGGCTTATATCTTCGATATCATCCAA-3' and AS5'-GGGGACCAGTTTGTACAAGAAAGCTGGGTGAATGATTAGTTTATGAGAGA-3'); At2g42940 (S5'-GGGGACAAGTTTGTACAAAAAAGCAGGCTCAGCAACTCTGACAGGCACC-3' and AS5'-GGGGACCAGTTTGTACAAGAAAGCTGGGTTGTTATGAATGTTGTTATATG-3'); At1g07370 (S5'-GGGGACAAGTTTGTACAAAAAAGCAGGCTTCAACAAAGTCAAACATACAGAAG-3' and AS5'-GGGGACCAGTTTGTACAAGAAAGCTGGGTTTTCGTCTTAGATATTATCAG-3'); At1g27900 (S5'-GGGGACAAGTTTGTACAAAAAAGCAGGCTACAAAATCAAGTGGGTTATTC-3' and AS5'-GGGGACCAGTTTGTACAAGAAAGCTGGGTATCGCAGAGAACACTCAAAGAACC-3'); At1g33430 (S5'-GGGGACAAGTTTGTACAAAAAAGCAGGCTTGTAATCATATGTTTTAGAAGC-3' and AS5'-GGGGACCAGTTTGTACAAGAAAGCTGGGTCTCCGCGCCTTAGTGC-3'); At5g07550 (S5'-GGGGACAAGTTTGTACAAAAAAGCAGGCTGTATGCATGCGCACACAAGCC-3' and AS5'-GGGGACCAGTTTGTACAAGAAAGCTGGGTTGGTGGGAAGAAGTGGGG-3'); At1g19070 (S5'-GGGGACAAGTTTGTACAAAAAAGCAGGCTCGTGAACAAAGGATTACG-3' and AS5'-GGGGACCAGTTTGTACAAGAAAGCTGGGTAACACAAACCCCCTTTCT-3'). Binary vectors were transformed into *Agrobacterium tumefacieriens *strain *gc101*. A previously described dip method [41] was utilized to develop transgenic *Arabidopsis*, as selected on Kanamycin. Two to five independent T1 plants bearing each GUS: Promoter construct were subjected to histochemical analysis for GUS activity. Transgenic and wild-type floral tissues were infiltrated using two vacuum pulses at 7 min each in GUS assay buffer (H_2_O, 0.1 M NaH_2_PO_4_, 10 mM Na_2_EDTA,. 0.5 M K_3_Fe(CN)_6_, 0.1% Triton X-100 and 0.3% 5-bromo-4-chloro-3-indoxyl-beta-D-glucuronide (X-Gluc) and incubated at 37°C for 12 h. Chlorophyll de-staining was performed with consecutive ethanol washes at 22°C. GUS stained regions were identified and fixed. Photographs were captured using an AxioVision digital camera (Zeiss) and compiled with the GNU Image Manipulation Program GIMP.

To construct the promoter: GUS vectors corresponding to ovule-specific candidate genes, promoter fragments were amplified by PCR using specific primers for each gene and inserted into a pBI101 plasmid after digestion with *BamH*I and *Hind*III (At1g27330 and At5g24420), *Hind*III and *Sal*I (At1g05550), or *Sal*I and *BamH*I (At5g49180 and At2g47470). Primers used for fragment amplification are: At1g27330 (S5'-GCAAGCTTGGACAGGGAAGAGAGCAT-3' and AS5'-GCGGATCCACTAGTTGCGGTCCTGAT-3'); At1g05550 (S5'-GCAAGCTTGATTGGGCCATCTCTTTTC-3' and AS5'-GCGTCGACGCTCCACCTCATCTTGAAGG-3'); At5g49180 (S5'-GCGTCGACTGGGTTTTGTTTCCTTCAGTG-3' and AS5'-GCGGATCCGTTGAACTCTCCGAGAAGG-3'); At5g24420 (S5'-GCAAGCTTGTCGTCGTCAGAGACCTTG-3' and AS5'-GCGGATCCTCTCATCGACCCAAAAGA-3'); At3g06240 (S5'-CAAAGAGCGAATTTCTCGGCTAC-3' and AS AS5'-TCTCTTGGAATCTCCGGTAGTTG-3'). For plant transformation, vectors were transferred into *Agrobacterium tumefaciens *strain ASE [65]. Transformations were performed on wild-type *Landsberg erecta *or *Col-0 *by floral dip transformation [64]. Seedlings obtained from the T1 promoter: GUS transformants were selected in MS medium with 50 μg/ml kanamycin. Dissected gynoecia were processed for GUS histology following [66]. Microscopical observations were conducted with a DRM Leica microscope and Nomarsky optics.

### Reverse transcription PCR validation experiments

cDNA synthesis was performed using 5 μg of total RNA and Superscript II Reverse Transcriptase (Invitrogen); reverse transcription PCR conditions were: 1 μg of cDNA, 1 mM forward and reverse primers (see below), 0.6 units Taq DNA polymerase (Invitrogen), 1 mL 10× PCR Buffer (Invitrogen). Primers used were: At4g04620 (S5'-AAGAGTTCCCGTGATTGTGG-3' and AS5'-AACCCGTCTTCGTCTTTGTG-3'); At1g19240 (S5'-CCTAATTGATCGGCCAGAAA-3' and AS5'-GACAAAGAAAACAGCGCACA-3'); At4g27860 (S5'-GGTGAGGAACCGAGCATAGA-3' and S5'-AGAGAAGCAACACCGCAGAT-3'); At4g14420 (S5'-TTGTCTTTGGCAGCTCATT-3' and AS5'-CCTGAGTTGCCTACCGTGTT-3'); At2g42710 (AS5'-AAGACGCAAAAGCTGGACAT-3' and 5'-GGATAACCCTTTCCCATCGT-3'). Amplification included 30 cycles of 94°C for 30 seconds, 60°C for 30 seconds, and 72°C for 32 seconds.

## Authors' contributions

Bioinformatics analyses, and Gateway Promoter constructs were performed by JP, with contributions in the earliest stages by HG. Promoter:GUS fusion validations and manuscript preparation were performed by JP. *In situ *hybridizations were performed by SK, and HS. Experiments and analysis of the ovule results were performed by MA-V, NS-L, and JPV-C. HS and BCM developed the experimental concepts and designed the experiments. BCM assisted in the writing of the manuscript.

## Supplementary Material

Additional file 1 **Spatially dissected Arabidopsis accessions and respective expression levels**. An excel spreadsheet (.xls) containing a sorted list of accession numbers of genes filtered for floral organ-enriched expression with their description and transcription levels in floral, leaf, and root libraries. Also included are data for determining the correspondence with prior microarray analyses and p-values for determining significant expression differences relative to wild type tissue and their relative over or under expression. Click here for file
